# The double-edged sword of online access to work tools outside work: The relationship with flexible working, work interrupting nonwork behaviors and job satisfaction

**DOI:** 10.3389/fpubh.2022.1035989

**Published:** 2023-01-26

**Authors:** Martina Hartner-Tiefenthaler, Ahmed Mohammed Sayed Mostafa, Sabine T. Koeszegi

**Affiliations:** ^1^Institute for Management Science, TU Wien, Vienna, Austria; ^2^Leeds University Business School, University of Leeds, Leeds, United Kingdom; ^3^Faculty of Commerce, Assiut University, Assiut, Egypt

**Keywords:** work connectivity behavior, online access to work tools outside work, flexible working, blurred boundaries, work interruption nonwork behaviors, job satisfaction

## Abstract

**Introduction:**

Information and communication technologies (ICTs) provide employees with *online access to work tools outside work* (OAWT), which can be seen as a double-edged sword fostering positive as well as negative aspects of flexibility. In our study, we investigated how OAWT relates to different forms of flexible working, work interrupting nonwork behaviors and job satisfaction.

**Method:**

We used a randomized sample of 758 Austrian employees from a broad range of organizations and tested the hypotheses by means of structural equation modeling.

**Results:**

Our findings revealed that OAWT is associated with available flexibility which relates positively to job satisfaction. However, at the same time, it is associated with required flexibility which relates negatively to job satisfaction and positively to work interrupting nonwork behaviors. OAWT has also been found to strengthen the positive relationship between required temporal flexibility and work interrupting nonwork behaviors, and attenuated the negative relationship between required temporal flexibility and job satisfaction.

**Implications:**

We discuss the practical implications and develop recommendations on how organizations should deal with OAWT.

## 1. Introduction

With the spread of COVID-19, it was impressively visible how employees' *online access to work tools outside work* (OAWT; e.g., having access to e-mails or online calendar outside work) helped with the organization of work in numerous industries. Many employees had to work from home, which resulted in odd work hours and access to work outside work hours (1). However, ubiquitous access to work is also accompanied with negative side effects such as mental health deterioration, which was highly likely during the pandemic (2, 3). Since working from home during the pandemic was not an autonomous choice, it is important to investigate the relationship of flexible working and boundary management without the omnipresent fear of COVID-19 (4, 5).

Even prior to the COVID-19 pandemic, information and communication technologies (ICTs) enabled employees to access digitalized information and data storages via mobile devices (6) and work, in particular knowledge work, was carried out anywhere and anytime (7). In general, being able to access work outside work hours grants flexibility, but also increases the likelihood that supervisors ask for availability at odd times (8, 9). Thus, work interrupts nonwork behaviors outside work hours, and flexible working seems to creep into employees' daily practice even without defining it as such. Work interrupting nonwork behaviors, therefore, represent an informal form of flexible working that might not be considered as such (e.g., a call from a colleague is not tracked in the time sheet). Traditionally, flexible working arrangements such as flexitime (i.e., available temporal flexibility) and telecommuting (i.e., available spatial flexibility) are implemented for employees to reconcile paid work and family demands (10, 11). However, being able to work flexibly outside the office also provides ubiquitous access to work even outside work hours. Thus, OAWT might encourage work interrupting nonwork behaviors since employees answer e-mails while commuting or during employees' designated nonwork time (12, 13). Although work interrupting nonwork behaviors have been found to positively relate to work-family conflict (14), there are employees, who favor the integration of both work and nonwork spheres over separating them (15, 16) as it provides flexibility (17) and enables them to individually craft work time and space (18). Nonetheless, integrating work and nonwork bears the risk of impairing workers' wellbeing due to lacking mental detachment from work during private time (13, 19–21).

Individual workers, organizations, and legislation actively need to address this behavioral contemporary phenomenon. Following the requirement that organizations are responsible to protect employees' health and introduce measures ensuring workers' safety and health at work (22), some organization such as Volkswagen have opted for blocking OAWT (23). Up to date, research dealing with this phenomenon mostly investigates the behavioral level. Knowledge about structural prevention measures (e.g., blocking OAWT) are mostly lacking although generally structural prevention measures are considered as more favorable than behavioral prevention measures (24). Therefore, this paper aims to provide an analysis about how OAWT relates to flexible working, work-interrupting nonwork behaviors, as well as job satisfaction. The focus on job satisfaction is mainly because it is widely regarded as the “most focal” employee attitude from the viewpoints of both research and practice and has been found to be strongly related to a wide range of desirable employee outcomes, such as improved job performance, increased life satisfaction and reduced withdrawal behaviors (25) and is associated with mental as well as physical health (26). Therefore, identifying its antecedents is of utmost importance.

**Figure 1:**
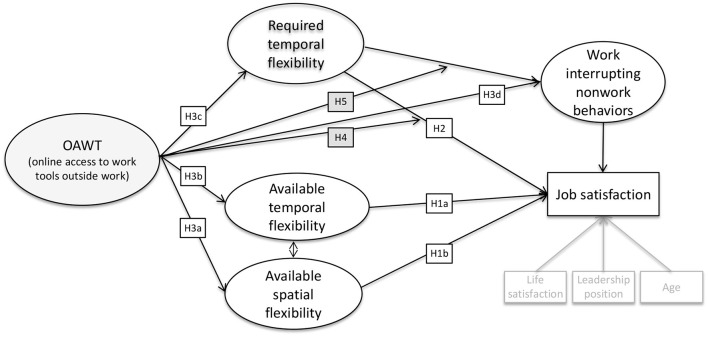
Overview of research model.

To summarize, OAWT can be seen as a double-edged sword providing positive as well as negative affordances and requiring employees to actively manage interruptions from both the work and the nonwork spheres. Since a growing number of jobs and work tasks allow or require flexibility (17, 27), we argue that studies about flexible working need to take into account work interrupting nonwork behaviors. We connect two different research streams: the literature on flexible working and the blurred boundaries between work and nonwork literature. Only recently, due to the COVID-l9 pandemic, both research streams have been started to be researched jointly (5). Both, however, require OAWT and, therefore, we investigate how OAWT shapes flexible working and the relationship with work interrupting nonwork behaviors and job satisfaction (see Figure 1). Going beyond previous research in this field, we explore the particular role of ICT and investigate the *access provided* by technology, *not the usage of* technology (28) to derive knowledge about structural parameters relating to different forms of flexibility as well as work interrupting nonwork behaviors and job satisfaction. This enables a more inclusive perspective on contemporary working (29–32), and allows deriving guidelines for organizations and employees on how to deal with ICT-enabled work extension.

## 2. Flexible working and job satisfaction

In several organizations, there is a debate about blocking employees' e-mail access outside work (23, 33). Despite its positive effect toward more flexibility (34), endless connectivity to the workplace might impair employees' ability to detach from work while outside of work (35), potentially impairing employee wellbeing and health (5, 13, 20). In this section, we shed light on both positive as well as negative consequences of flexibility and discuss how the bright and the dark sides of flexible working could be related to job satisfaction. Figure 1 summarizes the proposed relationships.

### 2.1. The bright side of flexible working: Available flexibility

Flexible working encompasses *temporal flexibility* (36) referring to the variation in the number of hours worked and the timing of work as well as *spatial flexibility* referring to work outside employers' premises connected to an office infrastructure which often takes place at employees' homes (37). Research about flexible working and job satisfaction rarely considers both temporal and spatial flexibility simultaneously (38). Often, flexible working is in place to reduce negative and stressful spillovers from work to private life (39). This potential for increased reconcilability of work and nonwork is also appreciated by employees and research unequivocally shows that employees' possibility to decide when and where to work positively relates to job satisfaction (40, 41). In fact, regardless of whether the organization formally or informally provides flexible working to its employees, the positive relationship between flexible working and job satisfaction remains (42).

Flexible working enables employees to autonomously decide when and where to work and, thus, grants employees more autonomy and control in their jobs. The job-demands-resources model (43), commonly referred to in work psychology, considers autonomy as a job resource that triggers motivation and satisfaction. In line with that, a lot of studies show the positive relationship between autonomy and psychological wellbeing (44–48). Thus, we hypothesize that the availability of flexible working positively relates to job satisfaction. Following Allen and colleagues (38), we distinguish between temporal as well as spatial flexibility.

**Hypothesis 1**. *Available (a) temporal and (b) spatial flexibility relate positively to employees' job satisfaction*.

### 2.2. The dark side of flexible working: Required temporal flexibility

In addition to employees' flexibility via ICTs, mobile technology users also perceive pressure to stay available and connected to work (5). This paradoxical situation was coined as the “empowerment-enslavement paradox” (49). Similarly, the “autonomy paradox” (50) describes once employees are able to respond to emails outside the office, their usage of mobile e-mail devices leads to an escalation of work-related e-mail behavior over time and they deliberately increase work beyond work hours due to the felt obligation to be available. Despite employees' autonomy to decide on the time to use their email devices for professional communication, they end up using it all the time, which actually diminishes their autonomy in practice. Thus, the autonomy paradox reflects the presence of tension between employees' personal autonomy and felt obligation toward the job and colleagues. Furthermore, when e-mails are regularly answered outside work hours, expectations of fast responses emerge leading to lower autonomy on the long run.

The critical perspective of workplace flexibility is also rooted in sociological work (51) addressing potential risks of the transformation toward increased workplace flexibility. We address the risk of felt obligations to be available *via* ICTs outside work hours (8) and investigate *required temporal flexibility*, which refers to employees individually perceived expectations concerning their flexibility in time (52–54). Consequences of workplace flexibility should vary depending on whether the control lies within the employer or the employee (55). Thus, we investigate the relationship between employees' required temporal flexibility and their job satisfaction. Following the job-demands-resources model (43), we consider the requirement as job demand and propose that job satisfaction is impaired when employees are required to be flexible in work time since employees' autonomy is limited.

**Hypothesis 2**. *Required temporal flexibility relates negatively to employees' job satisfaction*.

### 2.3. OAWT relates to the bright and dark side of flexible working

Empirical studies dealing with professional ICT usage either draw on qualitative research methods (34, 49, 50) or investigate the behavioral aspects of ICT-enabled work extension (1, 4, 13, 21). But showing this behavior and responding to professional e-mails outside work, first, requires the structural condition to being able to access work. This possibility was provided *ad-hoc* for many employees during the COVID-19 pandemic for working from home. However, which side effects accompany the technological possibility to access work from anywhere? In contrast to previous studies, we focus on the *access* provided by ICT and not on the actual usage of ICTs and investigate how different forms of flexibility relate to employees' online access to work tools outside work (i.e., OAWT).

OAWT has been controversially discussed (13, 52, 56): On the one hand, OAWT provides autonomy and flexibility for employees as it enables them to access work independently from their work hours and working place (7, 28). But on the other hand, following the autonomy paradox (50), employees' professional use of ICT devices outside work fosters a perceived obligation of availability over time. This encourages employees to be available for work purposes also outside work hours leading to the interruption of nonwork behaviors and recovery from work. Thus, we consider OAWT as double-edged sword fostering positive as well as negative aspects of flexibility.

**Hypothesis 3**. *OAWT relates positively to (a) available spatial flexibility, (b) available temporal flexibility, (c) required temporal flexibility, and (d) work-nonwork interruptions*.

### 2.4. OAWT moderates the dark side of flexibility

OAWT enables employees to deliberately decide when to answer e-mail requests. Therefore, we argue that the high convergence of work time and workplace due to OAWT also provides some form of boundary control. The boundary between work and nonwork is described in boundary theory (57). This boundary must be crossed each workday and can be marked by physical (i.e., specific settings), temporal (i.e., working times) as well as psychological aspects (i.e., role identities). Boundary control is defined as “psychological interpretations of perceived control over one's boundary environment” (14) and was found to mediate the negative relationship between ICT demands and rumination (58).

The job-demands-resources model (43) explains how job resources such as autonomy buffer the detrimental effects of high job demands (59, 60). Thus, we assume that the negative relationship between required temporal flexibility and job satisfaction might be attenuated as OAWT provides more autonomy for employees about their boundary between work and nonwork.

**Hypothesis 4**. *OAWT attenuates the negative relationship between required temporal flexibility and job satisfaction*.

Over time, perceived obligations of availability outside work emerge merely due to the possibility to access it also outside the office (50). Thus, OAWT results in behavioral consequences such as work interrupting nonwork behaviors. Work-nonwork boundary management strategies (14) build on Greenhaus and Beutell's (61) behavior-based “work-family conflict” model, but do not contain an evaluation of whether the interruptions cause any strain or not (14) as some employees prefer the integration of work and nonwork over separating both spheres (15, 16). Thus, work interrupting nonwork behaviors can be seen as a behavioral consequence of the possibility and/or requirement of flexible working.

Since we are particularly interested in how OAWT moderates the dark side of flexibility, we aim to shed further light on how OAWT shapes the direct relationships between required temporal flexibility and work interrupting nonwork behaviors as well as job satisfaction. Generally, requirements from work might interrupt nonwork activities due to the need for temporal flexibility even when employees *do not* have OAWT. For example, employees might be asked to work longer hours or change their work hours on short notice. However, due to the affordances of ICT to easily engage in *ad hoc* boundary crossings between work and nonwork, temporal requirements might have become independent from the workplace and thus, the restraining factor of geographical distance in order to accomplish the perceived obligation vanishes. Therefore, the physical boundary from nonwork to work does not need to be crossed in order to behaviorally act in the work role (62) and employees' additional effort for work interrupting nonwork behaviors is lower when OAWT is available. Also drawing on the autonomy paradox (50), we expect that OAWT strengthens the assumed positive relationship between required temporal flexibility and work interrupting nonwork behaviors.

**Hypothesis 5**. *OAWT strengthens the relationship between required temporal flexibility and work interrupting nonwork behaviors*.

## 3. Method

### 3.1. Sample

In Austria, each employee is an obligatory member of the Chamber of Labor. We used the Chamber's member list from selected industries in Lower Austria (i.e., wholesale, information and communication, financial and insurance activities, management consultancy, architectural and engineering activities, scientific research and development, and advertising and market research, education). The Chamber of Labour of Lower Austria randomly selected 10,106 employees and sent out paper-pencil questionnaires including return envelopes (postage paid upon reception). We opted for a paper-pencil study to avoid selection bias based on e-mail access. In total, 757 questionnaires were returned. This represents a response rate of 7.5 percent. Although this response rate seems to be very low at first sight, one must consider that many employees were invited, who have no affinity to research and maybe some of them even do not understand German. Our study, unlike most other studies in this area, addressed a wide spectrum of employees targeting the general workforce. Since the response rate is like other studies (63), we consider it as adequate.

Overall, the number of participants was balanced regarding gender (48% male). Nearly half of the participants (42%) had at least one child living in their households. Mean age was 39.97 years (*SD* = 10.84) and actual work hours (including overtime) amounted to 39.01 hours per week (*SD* = 10.04) on average. Regarding participants' education level, a third (34%) indicated to have a university or college degree and a further third (33%) a high school diploma as highest completed degree. More than a quarter of the participants (28%) were in a leadership position. On average, employees had been employed for 9.80 years (*SD* = 9.31) at their organization.

### 3.2. Non-response bias

Due to anonymous participation in our survey, we cannot compare our sample with non-respondents (i.e., members who were invited to participate, but did not respond). However, we can compare (very limited) basic socio-demographic values of our sample with the members who were invited. In line with existing research (64), the likelihood to participate in the survey was higher among women than among men. Although the percentage of men is higher in the working population (58%), as noted before, it is rather balanced in our sample (48%). Furthermore, the number of white-collar workers is considerably higher in our sample (90%) than in the group of employees invited (68%). Regarding age, our sample reflects the group of employees invited (*M* = 39). Overall, due to the randomized selection, this study also includes lower skilled employees, who potentially do not have OAWT and are generally under-researched in this area of research (65). We consider the randomized invitations as a strength because it enables us to attract employees who are underrepresented in research and in contrast to most of the studies in this area that rely on nonrandom samples, our results could be generalized.

### 3.3. Measures

Unless otherwise stated, all items were presented on 7-point Likert scales ranging from strongly disagree (1) to strongly agree (7). Table 1 provides all the items and their factor loadings.

**Table 1:** Factor loadings of each item.

	**λ**
***OAWT: online access to work tools outside work***	
Are you able to check your work-related e-mails outside of work?	0.88
Do you have access to your work-related calendar outside of work?	0.91
Do you have access to organization-specific data, applications or programs outside of work?	0.76
***Available temporal flexibility***	
I have the possibility to…	
Deliberately choose my daily work hours.	0.89
Schedule my work week myself.	0.86
Manage my own working time.	0.87
***Available spatial flexibility***	
I have the possibility to…	
Work from home instead of my usual workplace.	0.88
Assign myself where I do my tasks.	0.89
Be physically absent during meetings (telephone conference, video telephony, etc.).	0.56
***Required temporal flexibility***	
My work requires me to…	
Be flexible concerning my working time.	0.70
Work overtime.	0.71
Also work outside normal work hours.	0.90
***Work interrupting nonwork behaviors***	
I work during my vacations.	0.63
I respond to work-related communications (e.g., emails, texts, and phone calls) during my personal time away from work.	0.69
I regularly bring work home.	0.79

#### 3.3.1. Available temporal and spatial flexibilty

We used the German flexible working scale from Gerdenitsch et al. (66). Available temporal flexibility was assessed with three items (Cronbach's α = 0.90) focusing on employees' opportunity to autonomously decide when to work. Available spatial flexibility was also measured with three items (Cronbach's α = 0.81) asking about perceived autonomy to decide where to work and whether online participation in meetings is also allowed.

#### 3.3.2. Required temporal flexibility

We measured required temporal flexibility using three items from the German flexible working scale developed by Gerdenitsch et al. (66) (Cronbach's α = 0.81). The items describe employees' response to perceived organizational requirements regarding working time schedules (53).

#### 3.3.3. Frequency of OAWT

In order to capture OAWT, we developed a three-item scale (Cronbach's α = 0.81) assessing the frequency with which employees have access to their e-mails, work calendar, and work-related software/programs outside their work. Responses to the items ranged from never (1) to always (7). Although it is likely that most employees have OAWT either “never” or “always,” we opted for this answering format to have more information and capture varying access due to situations such as blocked e-mail access during specific times, lacking internet coverage at certain places, using a professional mobile internet device which is not used for private purposes, settings on the mobile phone, etc. As expected, the responses showed a bimodal structure toward the ends of the scale (*Md* = 5). To avoid information loss, we entered the 7-point values in the analysis but opted for an MLR (maximum likelihood estimation robust to non-normality) estimation, which, as the name suggests, is robust to non-normality.

#### 3.3.4. Work interrupting nonwork behaviors

The three items used are translated from the boundary management scale (14) to investigate work interrupting nonworking behaviors (Cronbach's α = 0.74). It captures employees' approach to demarcate their boundary between work and nonwork by assessing how often employees interrupt their nonwork behaviors due to work issues. Response options ranged from never (1) to always (7). For translating the items, we used Brislin's (67) back-translation method.

#### 3.3.5. Job satisfaction

We used a single item to capture a global rating of job satisfaction (“In general, how satisfied are you with your work?”). Using a single item for job satisfaction is considered a common and adequate procedure in work and organization research (47, 68–71) and has shown acceptable reliability and face validity (72, 73).

#### 3.3.6. Control variables

In organizations, flexible working is often provided to facilitate reconciability of work and family demands, in particular when children have to be cared for, which might be influenced by stereotypical gender roles. Thus, and in line with prior studies in this context (74, 75), we included the number of children in the household, gender (0 = male), age, actual weekly work hours, tenure, and leadership position as control variables in our analysis. Furthermore, the degree of flexible working is contingent on the type of job. To control for the influence of the nature of the job, we asked participants to indicate their job title. We then coded participants' job titles into seven categories based on the International Standard Classification of Occupations (ISCO) classification structure: Managers (*n* = 67), professionals (*n* = 67), technicians and associate professionals (*n* = 78), clerical support workers (*n* = 187), service and sales workers (*n* = 225), craft and related trade workers (*n* = 26), elementary occupations (*n* = 31), and plant and machine operators (*n* = 7). Finally, we also included life satisfaction due to its strong relationship with job satisfaction (76). In line with literature (77), life satisfaction was measured with one item on a 7-point scale asking “In general, how satisfied are you with your life?”.

### 3.4. Analysis

Following Anderson and Gerbing's (78) recommendations, we first assessed the validity and reliability of our measures using confirmatory factor analysis (CFA), and then tested the hypothesized relationships with structural equation modeling (SEM) using Mplus (Version 8) (79). We assessed model fit using the indices recommended by Williams, Vandenberg and Edwards (80). In particular, we used the comparative fit index (CFI), the root mean square error of approximation (RMSEA), and the standardized root mean square residual (SRMR). Good fit is attained when the CFI is above 0.95, the RMSEA is < 0.08, and the SRMR is < 0.10 (80).

One of the key limitations of traditional techniques of analysis of interaction effects, such as moderated regression with observed variables, is that these techniques suffer from low power because they do not control for explanatory variables measurement errors. Therefore, latent interaction modeling with SEM has been proposed by researchers as a better alternative (81, 82). A key advantage of using latent variables and SEM is the ability to control for different types of random and non-random measurement errors. This should in turn result in more accurate parameter estimates (81, 82).

#### 3.4.1. Testing validity and reliability of scales

The factor structures of all scales were tested using CFA. A good model fit was achieved [χ^2^ [131] = 386.36, *p* < 0.001, *RMSEA* = 0.06, *SRMR* = 0.05, *CFI* = 0.96]. The error terms for access to e-mails and calendar were correlated. It is reasonable that when having access to e-mails, the access to the work calendar is highly connected (as often the same mail programs are used for that). To check whether our proposed factor structure is the most likely one, we compared it with competing models such as two, three, four or five-factor models. Table 2 shows that our proposed six-factor model fits the data best.

**Table 2:** Measurement models comparison.

**Model**	**χ^2^(df)**	***Δχ* ^2^**	**CFI**	**TLI**	**RMSEA**	**SRMR**
Six-factor model (baseline model)	296.48 (89)	-	0.96	0.95	0.055	0.045
Five-factor model: combined available spatial flexibility and OAWT	523.90 (94)	227.42^***^	0.92	0.90	0.078	0.063
Five-factor model: combined available spatial and temporal flexibility	994.29 (94)	697.76^***^	0.83	0.78	0.112	0.083
Four-factor model: combined available spatial and temporal flexibility; required temporal flexibility and OAWT	1,523.71 (98)	1,227.23^***^	0.73	0.67	0.139	0.115
Three-factor model: combined available spatial and temporal flexibility; required temporal flexibility, OAWT and nonwork-work interruptions	1,553.25 (101)	1,256.77^***^	0.73	0.67	0.138	0.107
Two-factor model: all types of flexibility measurements including interruptions; job satisfaction	2,134.80 (103)	1,838.32^***^	0.62	0.55	0.161	0.118

The Δχ^2^is in relation to the baseline model; ^***^p < 0.001.

Job satisfaction was measured with a single item and thus the variance was fixed resulting in the same estimates of the two-factor model and the single-factor model.

The composite reliability scores for all constructs were above 0.75 and the average variance extracted was above 0.50 as can be seen in Table 3. Composite reliability is the extent to which a set of indicators share in their measurement of a construct (83). It is a measure of the homogeneity and internal consistency of the items that form a scale. Constructs that are highly reliable are the ones in which indicators are highly intercorrelated because this suggests that they are all measuring the same latent construct. Composite reliability values of 0.6 or more are generally regarded as acceptable (84). Therefore, all constructs in our study had high internal consistency (85). The square root of the average variance extracted of each construct exceeded the corresponding inter-construct correlations (85). Therefore, discriminant validity was also achieved.

**Table 3:** Means, correlations and reliability.

	**Items**	**M**	**SD**	**1**	**2**	**3**	**4**	**5**	**6**	**7**	**9**
1. Age	1	39.97	10.84								
2. Leadership position	1	0.28	0.45	0.20^***^							
3. OAWT	3	4.49	2.41	0.06	0.28^***^	0.85, (0.89)					
4. Available temporal flexibility	3	3.98	1.98	0.04	0.21^***^	0.45^***^	0.87, (0.91)				
5. Available spatial flexibility	3	4.45	1.79	0.02	0.26^***^	0.73^***^	0.64^***^	0.79, (0.83)			
6. Required temporal flexibility	3	4.45	1.79	0.07	0.28^***^	0.47^***^	0.23^***^	0.39^***^	0.78, (0.82)		
7. Work interrupting nonwork behaviors	3	3.14	2.24	0.06	0.28^***^	0.67^***^	0.31^***^	0.58^***^	0.61^***^	0.70, (0.75)	
9. Life satisfaction	1	5.87	1.15	0.05	0.08^*^	0.04	0.10^*^	0.05	−0.08	–0.11^*^	
10. Job satisfaction	1	5.63	1.31	0.13^***^	0.13^***^	0.04	0.23^***^	0.16^***^	–0.14^**^	−0.05	0.43^***^

M, Mean; SD, Standard deviation; Correlations are based on latent construct. Sub-diagonal entries are the latent construct inter-correlations. The first entry on the diagonal is the square root of the average variance extracted (AVE), while the second entry (in brackets) is the composite reliability. ^***^p < 0.001, ^**^p < 0.01, ^*^p < 0.05.

Since the measures for all variables were rated at the same time and by the same respondents, the likelihood of common method bias affecting the results is high. Therefore, we tested for common method bias using the latent method factor approach following the procedure described by Liang et al. (86). All items were allowed to load on their theoretical constructs and a latent method factor. Average variance extracted by the common method factor was 0.23, which is lower than the 0.50 threshold suggestive of a substantive construct (85). Accordingly, we conclude that common method bias was unlikely to be problematic.

#### 3.4.2. Hypotheses testing

We tested our hypotheses using MLR. The first model tested all associations indicated in Figure 1 without interactions (H1 to H4). The proposed structural model fit was good [χ^2^ [138] = 476.30, *p* < 0.001, *RMSEA* = 0.06, *SRMR* = 0.07, *CFI* = 0.94]. To keep the model as parsimonious as possible we excluded the non-significant control variables and retained life satisfaction (β = 0.39, *p* < 0.001), age (β = 0.10, *p* < 0.01), and leadership position (β = 0.08, *p* < 0.05) in the model. The more the respondents were satisfied with their life in general, the more they were satisfied with their jobs. The older the respondents were, the slightly higher they indicated to be satisfied with their jobs. Employees holding a leadership position were more satisfied with their job than employees who did not hold leadership positions. Overall, in this model, the predictor variables explained 26% of the variance in job satisfaction (*R*^2^ = 0.258).

The results about available flexibility were in line with H1a and H1b: Available temporal flexibility (β = 0.15, *p* < 0.01) and available spatial flexibility (β = 0.19, *p* < 0.01) both related positively with job satisfaction. In line with H2, required temporal flexibility (β = −0.20, *p* < 0.001) related negatively with job satisfaction. Required temporal flexibility also related to work interrupting nonwork behaviors (β = 0.37, *p* < 0.001). OAWT was associated with available temporal flexibility (H3a: β = 0.43, *p* < 0.001), available spatial flexibility (H3b: β = 0.74, *p* < 0.001), required temporal flexibility (H3c: β = 0.49, *p* < 0.001) and work interrupting nonwork behaviors (H3d: β = 0.51, *p* < 0.001), but not with job satisfaction (β = −0.13, *p* = 0.10). A positive indirect relationship of OAWT on job satisfaction was found via available temporal flexibility (β = 0.06, *p* < 0.01) and available spatial flexibility (β = 0.14, *p* = 0.01) and a negative indirect relationship of OAWT on job satisfaction was found via required temporal flexibility (β = −0.10, *p* < 0.001). Table 4 provides an overview of the results.

**Table 4:** Model testing results.

	**β**	**SE**
Age -> job satisfaction	0.104^**^	0.033
Leadership position -> job satisfaction	0.083^*^	0.034
Life satisfaction-> job satisfaction	0.387^***^	0.039
Available temporal -> job satisfaction flexibility	0.145^**^	0.049
Available spatial flexibility -> job satisfaction	0.185^**^	0.069
Required temporal flexibility -> job satisfaction	–0.204^***^	0.051
Work interrupting nonwork behaviors -> job satisfaction	0.024	0.068
OAWT -> job satisfaction	−0.126	0.077
OAWT -> required temporal flexibility	0.486^***^	0.038
OAWT -> available temporal flexibility	0.433^***^	0.039
OAWT -> available spatial flexibility	0.741^***^	0.025
OAWT -> Work interrupting nonwork behaviors	0.507^***^	0.042
Work interrupting nonwork behaviors -> required temporal flexibility	0.366^***^	0.042
Variance explained job satisfaction (*R^2^*)	0.258	
Variance explained required temporal flexibility (*R^2^*)	0.237	
Variance explained available temporal flexibility (*R^2^*)	0.187	
Variance explained available spatial flexibility (*R^2^*)	0.549	
Variance explained work interrupting nonwork behaviors (*R^2^*)	0.572	

SE, standard error; ^***^p < 0.001, ^**^p < 0.01, ^*^p < 0.05.

**Figure 2:**
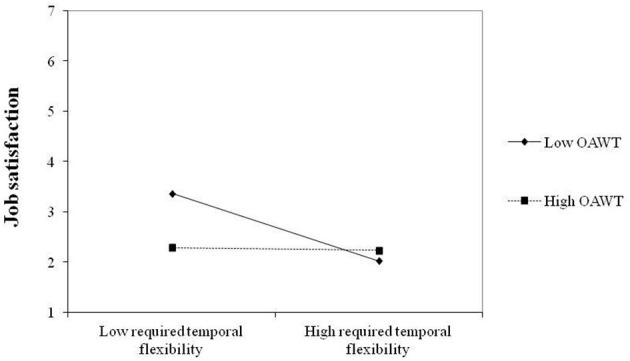
Latent interaction of required temporal flexibility and OAWT on job satisfaction.

In the second model, we tested whether OAWT moderates the relationship between required temporal flexibility and job satisfaction controlling for all other relationships indicated in the first model. The interaction effect testing H4 was significant (β = 0.09, *p* < 0.05). Once OAWT is taken into account, the negative relationship of required temporal flexibility on job satisfaction is attenuated (see Figure 2). The simple slope analysis shows that when OAWT is high (1 SD above the mean), the negative relationship of required temporal flexibility with job satisfaction becomes statistically insignificant (b = −0.015, *p* = 0.865) whereas when OAWT is 1 SD below the mean (b = −0.320, *p* < 0.001) and at the mean (b = −0.167, *p* < 0.001) the relationship between required temporal flexibility and job satisfaction was found to be negative. The predictor variables explain 26% of the variance of job satisfaction (*R*^2^ = 0.260) in this model.

**Figure 3:**
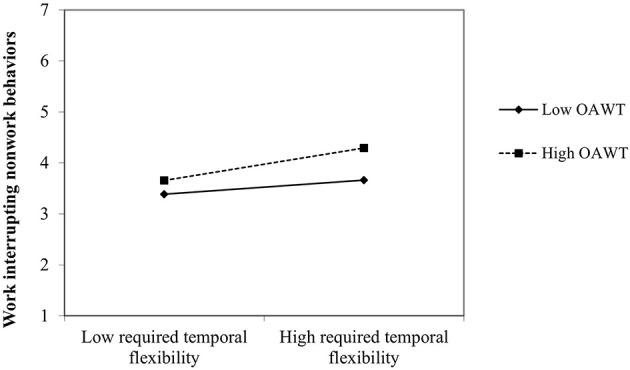
Latent interaction of required temporal flexibility and OAWT on work interrupting nonwork behaviors.

Finally, in the third model, we tested whether OAWT moderates the relationships between required temporal flexibility (H5) and work interrupting nonwork behaviors controlling for all other relationships indicated in the first model. Our results (see Figure 3) revealed an interaction effect (β = 0.31, *p* < 0.001). The simple slope analysis shows that when OAWT is low (1 SD below mean), the relationship between required temporal flexibility and work interrupting nonwork behaviors is negative (b = −0.109, *p* < 0.05) whereas when OAWT is high (1 SD above mean) the relationship between required temporal flexibility and work interrupting nonwork behaviors is positive (b = 0.567, *p* < 0.001). The predictor variables explain 74% of the variance of work interrupting nonwork behaviors (*R*^2^ = 0.736) in this model.

## 4. Discussion

In our study we reflect on the debate about blocking employees' e-mail access outside work (87) and investigated how OAWT relates to available and required flexibility, work interrupting nonwork behaviors and job satisfaction. By considering the role of OAWT and assuming positive as well as negative affordances, we connect the literature of flexible working with ICT-enabled blurred boundaries and contribute in several ways.

First, we bring together two streams of research which are traditionally studied separately. Due to the affordances given by contemporary ICTs, flexible working does not necessarily require formal arrangements (42) as employees can access their work also outside work. Traditionally, studies on flexible working refer to a concept (either researching schedule control or telecommuting), which does not incorporate modern ICTs and the changed nature of flexible working (7). Also, during the COVID-19 pandemic flexible working was not always an autonomous choice since remote work was obligatory for many workers in order to prevent contagion with the virus (5). Since the pandemic, the (non-) academic discourse has emphasized that flexible working increases the likelihood of blurred boundaries and work intensification (88). Thus, despite OAWT's advantages for work enabling employees for more flexibility, a threat of constant availability is likely. When employees respond to a quick professional question *via* e-mail outside their work hours and outside their office, work interrupts nonwork behaviors independent from the location. However, this does not have to be considered as negative in any case (89). With blurred boundaries between work and nonwork, the conceptualization of work itself undergoes a fundamental shift toward more flexibility (34) and the traditional conceptualization of flexible working needs to be re-thought. Future studies need to further investigate how much flexibility is positive and when does too much flexibility turn into detrimental (health) effects?

Second, studies about professional ICT-usage after work hours (35, 90, 91) emphasize the potential detrimental effect on wellbeing due to the lack of mental detachment from work. We extend the behavioral perspective of extent of usage and investigate the structural dimension to derive conclusions about how organizations should deal with OAWT. Our results revealed that OAWT can indeed be considered as a double-edged sword. OAWT is positively associated with the beneficial aspects of flexibility such as available flexibility as well as with the detrimental aspects of flexibility such as required flexibility. Thus, when implementing flexible working, it is crucial to consider potential negative aspects as well as the positive ones.

Third, we show how OAWT plays a role for the relationships on the “dark side of flexibility.” Only when OAWT is high, the relationship between required temporal flexibility and work interrupting nonwork behaviors is positive. When OAWT is low, the relationship is negative indicating that some perceived obligation might not be fulfilled on the behavioral level. This might suggest that blocking OAWT is a suitable measure for organizations to prevent employees from exhaustion. However, blocking OAWT would also limit employees' boundary control since our results also revealed that OAWT helps to cope with required temporal flexibility attenuating the negative relationship between required temporal flexibility and job satisfaction. When OAWT is provided, employees can deal with work issues from anywhere even though the work might not be executed within regular work hours. This aspect is under-emphasized in literature as most studies frame ICTs usage outside work (35) as being predominantly detrimental for employees. We draw attention to the–often neglected–positive role of work connectivity, which helps individuals cope with high demands of required temporal flexibility.

Fourth, by using a sample based on randomization from an exhaustive list of employees in Lower Austria, we distinguish ourselves from commonly used convenience samples. Although a self-selection bias toward higher education is likely, we were able to capture a considerable number of participants that could not access their e-mails outside their office or did not even have professional e-mail accounts reflecting the diversity in the workforce. Furthermore, since the data was collected prior to the pandemic, we can cancel out confounding with the need to work from home and being flexible because of private needs (e.g., homeschooling) or health restrictions (e.g., contagion with the virus).

### 4.1. Practical implications

The findings of our study have important practical implications related to the debate about blocking OAWT. Although OAWT is directly associated with potentially detrimental aspects of flexibility such as required temporal flexibility, it also relates positively to available temporal and spatial flexibility, which is seen as something beneficial. Results further suggest that having access to work anytime gives employees additional discretionary control over their work and buffers the detrimental impact of required temporal flexibility on job satisfaction. In more detail, our results revealed that when OAWT is high, the relationship between required temporal flexibility and work interrupting nonwork behaviors is positive whereas when OAWT is low, it is negative. Without OAWT, perceived requirements of temporal flexibility might not result in behaviors, but could nonetheless cognitively impact employees' wellbeing (e.g., by thinking on work and knowing that the task cannot be completed).

High work interrupting nonwork behaviors did not relate to low job satisfaction, which is in line with previous research (28, 89). We argue that sometimes it might be better to interrupt nonwork behaviors and finish a work task to satisfy the need for closure (92). This means that blocking employees' e-mail outside work hours may not be the optimal way to deal with employees' connectivity despite the potential downsides of OAWT. Blocking employees' OAWT impairs employees' autonomy (i.e., control). Thus, what seems to be important in organizations is the discussion of required temporal flexibility since the relationship with OAWT is rather high and required temporal flexibility relates negatively with job satisfaction. It is essential that organizations openly communicate their expectations regarding work-extending behaviors and thoroughly reflect their policies about employees' availability outside office hours.

### 4.2. Limitations

As in every study, this study is subject to several limitations. First, although a representative randomized sample was addressed, there is a self-selection bias. We tried to limit this bias by using paper-pencil questionnaires instead of online questionnaires. Despite this self-selection bias, we were able to use a highly diverse sample to increase generalizability of results.

Second, as in many empirical studies, the results are only based on a cross-sectional sample and no causal conclusions can be made. We tried to collect longitudinal data and asked respondents for their e-mail address for a follow-up study. Only 127 participants provided their e-mail address and were contacted 1.5 years later. Unfortunately, we could only match 25 data sets. Thus, we refrained from testing hypotheses with this limited longitudinal sample.

Third, we obtained a bimodal distribution for OAWT reflecting that many employees either constantly or never have OAWT although normal distribution is preferred in SEM. To deal with non-normality we used MLR estimation in m-plus which is robust to non-normality. Furthermore, the criteria for validity and reliability were also met and thus, we consider the measurement as appropriate. Furthermore, we consider the results about OAWT being the moderating variable as the most important ones, which should not be flawed by the bimodal structure (frequent access vs. no access).

Fourth, aiming to collect a sample that is highly representative, the data involving spatial flexibility is skewed. We had only a low number of employees in our sample who have the possibility for spatial flexibility which might be the reason that the positive relationship of available spatial flexibility on job satisfaction could not reach significance. This might be different nowadays, since many organizations were forced to provide home office to their employees in order to cope with the threats of COVID-19 (93). Thus, our analysis potentially underestimates the positive direct relationship between available spatial flexibility and job satisfaction.

## 5. Conclusions

Generally, we conclude that OAWT could be seen as a double-edged sword. Our results show that OAWT is associated with available flexibility that relates positively to job satisfaction. However, at the same time, it is also associated with required flexibility, which relates negatively to job satisfaction and positively to work interrupting nonwork behaviors. Our findings also revealed that, OAWT helps strengthen the positive relationship between required temporal flexibility and work interrupting nonwork behaviors. Yet, it also attenuates the negative relationship between required temporal flexibility and job satisfaction. Thus, it is important to critically reflect on the use of ICTs for work outside work hours. Even though OAWT strengthens the relationship between required temporal availability and work interrupting nonwork behaviors, it helps to cope with employees' required flexibility. This particularly needs to be considered when designing flexible working policies in organizations. Despite the general superiority of structural prevention measures over behavioral prevention measures, we highlight that blocking OAWT also reduces boundary control, which in turn, could impair employees' autonomy. Boundary control buffers the negative relationship between ICT demands and rumination of work after work hours (58). Thus, we rather suggest an open discussion about required temporal flexibility in organizations as blocking OAWT would also limit the positive effects of OAWT to cope with required flexibility.

## Data availability statement

The raw data supporting the conclusions of this article will be made available by the authors, without undue reservation.

## Ethics statement

Ethical review and approval was not required for the study on human participants in accordance with the local legislation and institutional requirements. The patients/participants provided their written informed consent to participate in this study.

## Author contributions

MH-T: planning and execution of the study, data analysis, interpreting results, writing—original draft preparation, and review and editing. AM: interpreting results, writing—original draft preparation, and review and editing. SK: writing—original draft preparation and review and editing. All authors contributed to the article and approved the submitted version.
